# Pathological Internet Use—An Important Comorbidity in Child and Adolescent Psychiatry: Prevalence and Correlation Patterns in a Naturalistic Sample of Adolescent Inpatients

**DOI:** 10.1155/2018/1629147

**Published:** 2018-03-29

**Authors:** Martin Fuchs, David Riedl, Astrid Bock, Gerhard Rumpold, Kathrin Sevecke

**Affiliations:** ^1^Universitätsklinik für Kinder und Jugendpsychiatrie, Medizinische Universität Innsbruck, Innsbruck, Austria; ^2^Universitätsklinik für Medizinische Psychologie, Medizinische Universität Innsbruck, Innsbruck, Austria

## Abstract

**Background:**

Few studies have examined the prevalence of problematic internet use (PIU) in young people undergoing inpatient treatment in child and adolescent psychiatry centers. The aims of our study were thus (a) to assess the frequency of comorbid PIU in a sample of adolescent psychiatric inpatients and compare it with a control group of nonreferred adolescents and (b) to gain insights into correlations between PIU and psychiatric comorbidities.

**Methods:**

111 child and adolescent psychiatry inpatients (CAP-IP, mean age 15.1 ± 1.4 years; female : male 72.4% : 27.6%) undergoing routine psychodiagnostics were screened for the presence of PIU. The widely used Compulsive Internet Use Scale (CIUS) was chosen for this purpose. Prevalence rates of PIU were then compared to matched nonreferred control subjects from a school sample. Additionally, comorbidities of inpatients with PIU were compared to inpatients without PIU.

**Results:**

Our inpatient sample showed a much higher prevalence of PIU than that found in previous populational samples of young people. Compared with a matched school sample, addictive internet use was 7.8 times higher and problematic internet use 3.3 times higher among our adolescent sample. PIU was significantly associated with characteristic patterns of psychopathology, that is, suicidality, difficulties in establishing stable and consolidated identity, and peer victimization.

**Conclusion:**

PIU among adolescents undergoing inpatient psychiatric treatment is much more frequent than among their peers in the general population and is associated with specific patterns of psychopathology.

## 1. Introduction

The use of the internet and related digital media has grown exponentially worldwide in recent years. Indeed, in mid-2016, a penetration rate of almost half of the world's population was recorded. This translates into a worldwide growth rate of more than 900% from 2010 to 2016. The fastest growing region was found to be the European Union, where in 2016, nearly 80% of the overall population was using the internet [1].

While this trend has been observed in all age groups, adolescents, in particular, adopt new technologies quickly. Virtually 100% of German-speaking adolescents use the internet on a daily basis, more than 80% use it on the go, and more than 50% consider it “indispensable” [2, 3]. Access to relevant technologies has become a precondition for educational and occupational advancement; thus, internet “abstinence” is not an option for the majority of youth today [2, 4].

For the past 20 years, the rising significance of the internet and associated media has stimulated debate about dysregulated or problematic use of the internet (PIU) among young people [5, 6] and about whether such dysfunctional patterns affect somatic health and psychosocial wellbeing [7].

From a pathophysiological point of view, PIU has been understood as an impulse control disorder that is best classified within the group of behavioral addictions. Despite a growing body of scientific evidence for this taxonomy [8–17], only “Internet Gaming Disorder (IGD)”—that is, the excessive use of online computer games, leading to functional impairment and distress—was included in Section III of the DSM-5 as a “condition that requires further study” [18].

Given the diversity of diagnostic assessment methods [19, 20], it is unsurprising that PIU prevalence figures vary substantially. The only meta-analysis to date included 80 studies with nearly 90,000 participants and a mean age of 18.4 years. The authors found mean worldwide prevalence of 6% for PIU, with the European subsample showing 4.35% [21]. In recent years, studies based on large representative samples of the European adolescent population have found PIU prevalence rates of 1.2–4.4% [4, 22–24]. Müller et al. investigated the occurrence of the proposed DSM-5 criteria for Internet Gaming Disorder (IGD) [18] in a large sample of more than 10,000 European adolescents, finding a prevalence of 1.6% [25]. In a representative sample of German adolescents, criteria for IGD were found in 1.16% of the study participants [26].

Our study used the Compulsive Internet Use Scale (CIUS), which has already been applied by several other investigations of German-speaking adolescents, yielding results comparable to ours. Rumpf et al. [27] found PIU to be prevalent in 2.4% of young people aged 14–24 years and in 4.0% of adolescents aged 14–16. In a second study, the authors found a prevalence rate of 3.2% in German adolescents aged 14–17 [24]. The only Austrian study to date using the CIUS [2] showed PIU to be prevalent in 3.3% of the sample (*n* = 398, mean age = 15.2 years). This school sample was used in the present study as a point of reference.

A growing body of evidence indicates that PIU is associated with a broad spectrum of somatic and psychosocial problems as well as with psychiatric comorbidity. Research on the negative somatic effects of excessive internet use in adolescents has found sleeping problems [8, 28–32], overweight [29, 33–35], poor nutrition [8, 36], and back and musculoskeletal issues [28]. With respect to psychosocial behavior, there is evidence that dysregulated internet use is associated with tobacco use [8], less time spent with real-life peers [37] and physical inactivity, or sedentary lifestyle [8, 38]. PIU has also been associated with self-destructive behavior in adolescents [9]. Two meta-analyses on internet addiction and psychiatric comorbidities in adolescents and adults showed comparable results. In these studies, PIU was associated with symptoms of ADHD, alcohol abuse, depression and anxiety, hostility and aggression, and obsessive-compulsive symptoms [39, 40]. In their large-scale and cross-national study on European adolescents (*n* = 11,356, mean age 14.9 years), Kaess et al. demonstrated that PIU in adolescents significantly correlated with suicidal behavior, depression, anxiety, conduct problems, and symptoms of ADHD [9]. The authors thus conclude that assessing PIU could be helpful for early detection and intervention in cases of suicidal ideation and psychopathology in adolescents. A close association between suicidality and PIU has also been reported by other studies [8, 41, 42].

In light of these findings, it is certainly worth exploring why PIU seems to play a role in such a broad spectrum of internalizing and externalizing psychopathology. In the case of internalizing comorbidity, PIU might be a “digital expression” or form of “digital compensation” of classical psychopathology symptoms, such as loss of enjoyable activities, loss of engagement with real-life people, social withdrawal, escapism, avoidance, loss of life satisfaction, and suicidality. In the case of externalizing comorbidity, the impulse control domain of disorders such as ADHD, borderline personality disorder, and NSSI may contribute to the association between these entities and PIU. The association between substance-use disorders and PIU is not surprising, given that they share pathophysiological mechanisms [12, 13, 16, 17]. It is important to emphasize that a potential causal relationship between PIU and psychiatric comorbidity is not yet clear because findings have primarily been based on cross-sectional data. Thus, conclusions on cause and effect cannot yet be drawn.

In adolescence, young people increasingly begin to establish social networks outside of the core family, and psychosocial development is strongly influenced by extrafamilial peers. The internet and social networking sites (SNS) can be an important and useful tool for young people to develop and experiment with relationships and social capital [43]. On the other hand, there is some evidence that in adolescents, intensive SNS use may be associated with poor psychological functioning, poor mental health, and suicidality, especially in the presence of cyberbullying [44, 45]. Instability in interpersonal relationships and problems with identity and ego strength may play a mediating role, but to our knowledge, this hypothesis has not yet been tested in studies.

Taken together, the “complex interaction between various aetiological factors” [40] and the ubiquitous availability of relevant technologies may have led to the high prevalence of PIU and associated psychopathology in adolescent populational samples. In recent European studies, PIU and associated psychopathology cases were equally distributed among male and female adolescents, in contrast to previous findings and popular belief [8].

Most findings on the comorbidity of PIU and psychiatric diagnoses in youth have been gathered in nonclinical samples. Few studies have examined adolescents undergoing inpatient psychiatric treatment. Müller et al. screened a sample of 81 juvenile patients and found a high prevalence of PIU, 11.3%, as well as an association with internalizing disorders such as anxiety and depression [46]. In another study, a sample of 60 referred adolescents with diagnosed PIU was screened for the presence of comorbid psychiatric disorders. The most common conditions found were ADHD, social phobia, and major depressive disorder [47].

To our knowledge, no study to date has directly compared the prevalence of PIU in adolescents undergoing inpatient CAP treatment with a matched control group of nonreferred peers using the same diagnostic instrument. The present study was thus conducted to test the following two hypotheses:

(1) Adolescents undergoing inpatient psychiatric treatment will show different rates of dysfunctional internet use (addictive internet use and problematic internet use) from a control group of nonreferred peers. Null hypothesis: the groups will not differ in terms of internet use.

(2) A group comparison of adolescent inpatients with dysfunctional internet use (addictive internet use and problematic internet use) and inpatients with normal use will show differences concerning additional clinical variables. Null hypothesis: the groups will not differ statistically in terms of other psychopathology.

## 2. Materials and Methods

### 2.1. Sample and Procedure

We analyzed data from inpatients treated in the Department of Child and Adolescent Psychiatry, Medical University of Innsbruck, between 2013 and 2017. The Department of Child and Adolescent Psychiatry is a specialized facility with a public service mandate for the Austrian federal state of Tyrol. It is the only hospital in the state with inpatient services for minors with mental health problems.

As part of a routine diagnostic battery, patients completed the Structured Clinical Interview for DSM-IV Axis II Disorders (SCID-II), the Youth Self-Report (YSR), and the Assessment of Identity Development in Adolescence (AIDA). A parent or legal guardian completed the Child Behavior Checklist (CBCL). Sociodemographic data was also collected at the beginning of inpatient treatment. For the purposes of this study, the Compulsive Internet Use Scale (CIUS) was added to assess PIU. The primary psychiatric diagnosis was extracted from electronic patient records.

Inclusion criteria were (a) minimum age of 12 years, (b) undergoing inpatient treatment in the Department of Child and Adolescent Psychiatry (Innsbruck), and (c) written declaration of consent. Exclusion criteria were (a) age below 12 years and (b) diagnosis of severe mental retardation or florid psychopathological symptoms (e.g., psychosis or intoxication), which would have made the completion of the questionnaires unfeasible. All patients and their parents or legal guardians signed an informed consent form for the scientific use of data. The study was approved by the Ethics Committee of the Medical University of Innsbruck and was performed according to the Declaration of Helsinki 1995 (as revised in Edinburgh in 2000).

### 2.2. Measurement

#### 2.2.1. CIUS

The Compulsive Internet Use Scale (CIUS) is a widely used self-report questionnaire with 14 items. It was developed to assess addictive internet use, on the basis of the criteria for substance dependence and pathological gambling in the Diagnostic and Statistical Manual of Mental Disorders, 4th Edition [48]. The 14 questions are answered on a 5-point Likert scale, resulting in a sum score between 0 and 56 points. A cut-off of 28 points identifies* pathological or addictive internet use* [49], while a cut-off of 21 points identifies a subthreshold level of* problematic internet use* [50]. The 14 items of the CIUS measure five subscales: loss of control, preoccupation, conflict, withdrawal symptoms, and coping. The CIUS has been validated in several languages, including German [51]. Good external and factorial validity as well as good reliability indices were reported for the questionnaire in samples of heavy internet users (Cronbach *α* = 0.89–0.90) and normative samples (*α* = 0.90) [49].

#### 2.2.2. Assessment of Identity Development in Adolescence (AIDA)

The Assessment of Identity Development in Adolescence (AIDA) self-report questionnaire was developed to measure fundamental subdomains of juvenile identity development. The instrument can distinguish between stable and consolidated identity at one end and identity diffusion at the other [52]. Identity diffusion is considered one of the core elements of borderline personality organization, but it is also part of the section “Criterion A: Level of Personality Functioning” of the alternative DSM-5 model for personality disorders [18]. The 58 items of the AIDA are summed up to a total score (extent of identity diffusion), which can also be divided into two subscales, discontinuity and incoherence. The instrument shows good reliability in total score (diffusion: *α* = .94), scale (discontinuity: *α* = .86; incoherence: *α* = .92), and subscale (*α* = .73–.86) as well as good validity [52–54].

#### 2.2.3. SCID-II

The Structured Clinical Interview for DSM-IV Disorders (SCID) is the official diagnostic instrument of the American Psychiatric Association (APA) for the assessment of psychiatric disorders via clinical interview. To diagnose personality disorders, the Structured Clinical Interview for DSM-IV Axis II Disorders (SCID-II) is used, which is available in German [55]. The German version is considered appropriate for the diagnosis of personality disorders in adolescence [56]. SCID-II consists of a self-report questionnaire followed by a clinical interview. Each part is scored as 0 (absent), 2 (subclinical), or 3 (present). Our calculations were based on the mean dimensional score (ranging from 0–3) of the three personality disorder clusters A, B, and C.

#### 2.2.4. CBCL and YSR

The Child Behavior Checklist (CBCL) and the corresponding Youth Self-Report (YSR) are among the most widely used instruments to assess mental health in minors [57]. Both instruments have been translated into more than 50 languages, and German versions have been available for approx. 20 years [58]. The CBCL features eight problem syndrome scales that can be combined into the broadband scales “Internalizing Behavior Problems” (Anxious/Depressed, Withdrawn/Depressed, Somatic Complaints), “Externalizing Behavior Problems” (Rule-Breaking Behavior, Aggressive Behavior), and “Mixed Problems” (Social Problems, Thought Problems, Attention Problems). A total behavior problems score (TOT) can also be calculated. Significant discrepancy between youth-reported and parent-reported psychopathology is a well-known phenomenon worldwide, being also evident in studies of large German samples [59]. For the CBCL and its subscales, good reliability (*r* > .86) and validity indices have been reported [57], with reliability indices for the YSR-subscales reported as sufficient (*r* > .70) [57].

#### 2.2.5. Primary Psychiatric Diagnosis

Electronic patient records were evaluated, extracting the primary psychiatric diagnosis according to ICD-10 [60]. For some calculations, we grouped inpatient diagnoses into the broader categories “internalizing disorders” (affective disorders and anxiety disorders) and “externalizing disorders” (hyperkinetic disorders and conduct disorders), which is common practice in child and adolescent psychiatry [61].

#### 2.2.6. (Cyber)bullying

As part of the routine clinical examination, patients were asked whether they had been bullied online or offline, and whether they were active bullies online or offline themselves. The total number of instances (being bullied online/offline, active bullying online/offline) was assessed for this study.

#### 2.2.7. Control Sample

To compare CAP-IP with nonreferred youth, we analyzed the data from a school sample from a previously published study [2]. This allowed us to compare two groups examined in the same state with the same instrument. The groups were matched for age and gender.

### 2.3. Statistical Procedures

Sample characteristics are given as frequencies, ranges, means, and standard deviations. IBM SPSS (version 22.0) was used for statistical analysis.

To compare the CIUS values of the inpatient sample with the control sample, independent sample *t*-tests and effect sizes (Cohen's* d*) were calculated. The odds ratio was estimated through binary logistic regression. Group differences were calculated for the overall samples and for age- and gender-matched comparisons. Twin-matching was conducted using the propensity score matching procedure.

The psychopathology of inpatients with PIU was compared to inpatients without PIU. Group comparisons were conducted using the chi-squared test or Fisher's exact test for nominal data and analyses of variance with sex as a covariate (ANCOVA) for interval data. Cohen's *d* was used to evaluate the effect sizes of the mean differences.

To investigate differences in internet use between the two groups, one-way analyses of variance (ANOVAs) with Bonferroni correction for post hoc comparison were calculated.

## 3. Results

### 3.1. Sample

Initially, 112 patients were included in this study. One patient was excluded due to missing CIUS data, thus yielding a final sample of 111 patients. The mean age was 15.1 (SD: 1.4) years, and the majority of patients were female (74.8%). 72.1% were still attending school, and about half of the sample were from families with separated or divorced parents. The majority of the patients had been diagnosed with an internalizing disorder (61.3%). About a fourth of the sample had previously tried to commit suicide, and 40.5% of the patients had had at least one episode of self-injury (see Table 1 for details).

Patients reported a mean score of 61.4 (SD: 26.8) on the YSR. 73.0% of the sample reported values above the clinical cut-off, with no gender differences (female: 73.0% versus male: 73.1%; *χ*^2^ = 0.01, *p* = 0.99). The large majority of the patients' parents (90.0%) described their children as being significantly distressed; the mean CBCL score was 58.5 (SD: 26.0).

As to PIU, the patients had mean values of 14.8 (SD: 13.8) on the CIUS, with 28.8% reporting values above the cut-off (8.1% problematic internet use, 20.7% addictive internet use). In terms of the subscales of the CIUS, mean values were the highest on the coping subscale and the lowest on withdrawal symptoms.

### 3.2. Comparison of the Clinical Sample with a Sample of Nonreferred Youth (School Sample)

To test our first hypothesis, we compared our clinical sample to a previously published sample of nonreferred adolescents (school sample, *n* = 398, mean age 15.2 ± 2.3, 34.2% female; see [2]). Based on CIUS cut-off scores, we categorized the two samples into* normal users*,* problematic users*, and* pathological users.*

**Figure 1:**
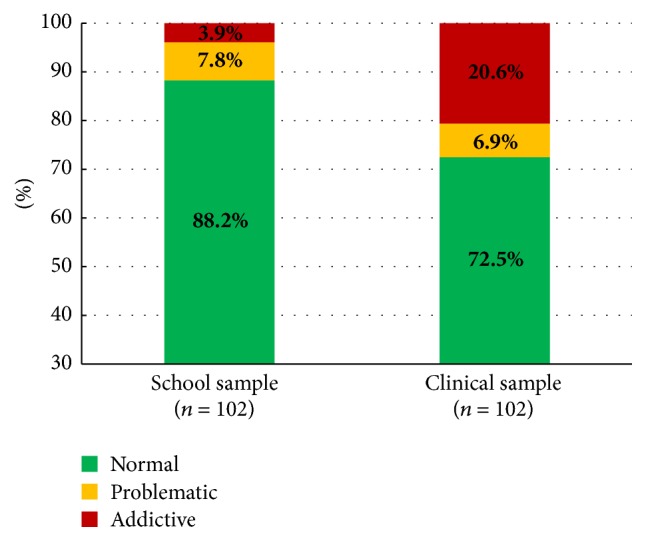
Internet usage (CIUS): comparison of school sample and clinical sample, age- and gender-matched (*n* = 204).

The results (see Table 2 and Figure 1) show that our patients reported significantly higher CIUS total values than the adolescents in the control sample (14.8 versus 11.2), with an effect size of* d *= 0.32. Addictive internet use (20.7% versus 3.4%; odds ratio: 7.0) and problematic internet use (28.8% versus 11.0%; odds ratio: 3.9) were significantly more prevalent in the clinical sample than in the control sample.

After twin-matching (age and gender) the two groups, there was still a significant difference between patients and control group: among inpatients, the probability (OR) of addictive internet use was 7.8 times higher and the probability of problematic internet use 3.3 times higher than their adolescent peers in the community (see Figure 1).

Our results therefore indicate that the null hypothesis may be rejected, as adolescents undergoing inpatient psychiatric treatment showed significantly higher rates of dysfunctional internet use (addictive internet use and problematic internet use) than a control group of nonreferred peers.

### 3.3. Analysis of the Clinical Sample: PIU versus Non-PIU

To test the second hypothesis, we divided the clinical sample into normal users (non-PIU, 71.2%, *n* = 79) and pathological or problematic users (PIU, 28.8%, *n* = 32) based on CIUS cut-off scores. The PIU group consisted of users with* pathological *or* addictive internet use *(cut-off: 28 points) and a subthreshold group with* problematic internet use* (cut-off: 21 points). All inpatients with a CIUS value below 21 points were considered* normal users*.

#### 3.3.1. Sociodemographic Data

Neither age nor family status was significantly associated with the incidence of PIU. Male patients showed higher prevalence rates of PIU (39.3% versus 25.3%), but this difference was not statistically significant (*p* = 0.23, see Table 3).

#### 3.3.2. Clinical Data

Patients with PIU showed significantly more suicide attempts than patients without PIU (46.9% versus 13.9%; *p* < 0.001). With regard to clinical diagnosis, patients with PIU showed more internalizing disorders (71% versus 57%), but this difference was not statistically significant. A small but statistically not significant difference was also found for NSSI, with patients with PIU showing higher rates (46.9% versus 38%; see Table 4).

#### 3.3.3. Psychometric Data (AIDA, SCID-II, YSR, CBCL)

Patients with PIU reported a mean score of 123.6 points on the AIDA total score, which was significantly higher than the mean score of patients without PIU (104.1; *p* = 0.005,* d *= 0.5), representing a moderate effect size. The analysis of the two subscales showed that patients with PIU had significantly higher scores on both the coherence subscale (68.1 versus 56.1; *p* = 0.004,* d *= 0.5) and the discontinuity subscale (55.5 versus 48.0; *p* = 0.021, see Table 5).

The SCID-II showed a significant group difference between PIU and non-PIU for cluster B personality disorders (*p* = 0.036,* d* = 0.5), whereas there was no significant group difference for cluster A or cluster C (see Table 5).

While patients with PIU reported significantly higher scores for attention problems than those without PIU (YSR: 8.8 versus 6.8; *p* = 0.043,* d *= 0.5), the ratings of parents of patients with PIU differed only on the withdrawn/depressed subscale (CBCL: 9.4 versus 6.9; *p* = 0.001,* d *= 0.8). All other subscales and broadband scales of the YSR and CBCL as well as the total behavior problems score (TOT) showed no statistically significant difference between patients with and without PIU (see Table 5).

#### 3.3.4. Peer Victimization

Patients with PIU reported significantly more instances of being bullied than patients without PIU, both offline (12.8 versus 7.0, *p* = 0.002,* d *= 0.7) and online (2.9 versus 0.9, *p* = 0.002,* d *= 0.8). Only 9 patients in the total sample (7.8%) stated that they had actively bullied other people offline. In our sample, there was no group difference between PIU and non-PIU patients regarding active online or offline bullying (see Table 6).

Our results indicate that for the second hypothesis, the null hypothesis may also be rejected: in terms of other psychopathology, we found significant differences between adolescent inpatients with dysfunctional internet use (addictive internet use and problematic internet use) and inpatients with normal use.

## 4. Discussion

Our study sought to address two issues: first, whether the prevalence of comorbid PIU in a naturalistic sample of inpatients in a specialized center for child and adolescent psychiatry would differ from a control group of nonreferred adolescents; second, whether adolescent inpatients with dysfunctional internet use and inpatients with normal use show differences concerning other psychopathology.

As the data show, the prevalence of addictive internet use (20.7% versus 3.4%; odds ratio: 7.0) and problematic internet use (28.8% versus 11.0%; odds ratio: 3.9) was significantly higher in the clinical sample than in the control sample, thus confirming the first hypothesis. The differences remained stable after twin-matching the two samples. As described in detail in a previous publication of our group [2], prevalence rates of addictive and problematic internet use in the school sample show high consistency with European large-scale studies [4, 22–24, 27]. This may be indicative of the representativeness of our control sample and of the broader generalizability of our findings. To our knowledge, only one other study [46] has examined addictive internet use as a comorbid disorder among adolescent psychiatry patients, finding a prevalence of 11.3%. In our study, which used a different instrument, 20.7% of the inpatients showed addictive usage patterns. The rise of mobile internet use in recent years may have contributed to this.

Nearly 30% of all adolescent inpatients in our study showed signs of dysfunctional internet use. In clinical practice, therefore, digital media use should be taken into consideration on a routine basis in inpatient treatment and assessment. Our data suggest that young people with mental health issues have difficulties acquiring balanced, competent media use skills.

**Figure 2:**
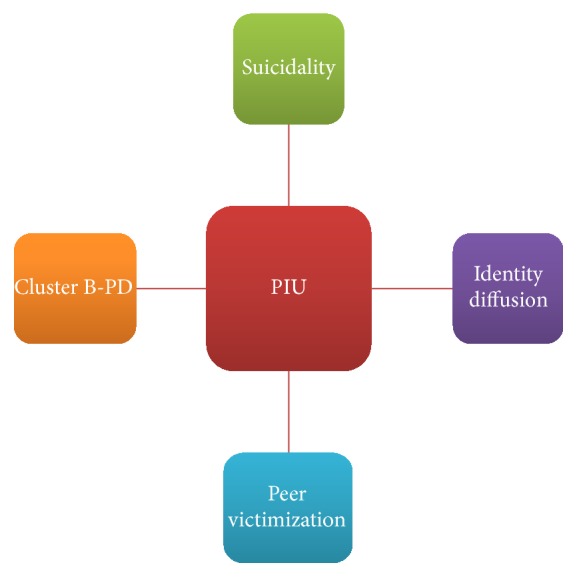
Cluster of psychopathology associated with PIU.

As to our second hypothesis, data from this study show that within the inpatient group, adolescents showing signs of PIU differed significantly from normal users in several psychopathological features.  Figure 2 shows these associations schematically.

First, patients with PIU had significantly more suicide attempts than patients with normal internet use. This finding is in line with several other studies showing a close association between suicidality and PIU [8, 9, 41, 42]. Second, patients with PIU reported more difficulties in establishing a stable and consolidated identity and especially problems with ego strength, suggestibility, awareness of a defined core and inner substance, and understanding motives and behavior. To our knowledge, this study is the first to demonstrate an association between juvenile PIU and identity problems using a structured questionnaire (AIDA). A third association was found between PIU and cluster B personality disorders, especially juvenile borderline personality disorder. To our knowledge, there are no other studies showing this association in adolescent inpatient samples. One study found similar associations in a French adult sample of outpatients [62]. The association of identity diffusion with aspects of personality disorders in our PIU sample indicates that this psychopathological cluster specifically interferes with the consolidation of competent media use. Potentially, instability in interpersonal relationships and problems with identity and ego strength are expressed or compensated in these adolescents via online outlets. Fourth, patients with PIU reported significantly more instances of being bullied than patients without PIU, both offline and online. This is in line with several studies showing this association in adolescent European [63] and Asian [64] samples. In our study, being bullied online (“cyberbullying”) was the far less frequent scenario than being bullied “face to face”, in both the normal use group and the PIU group. This supports the findings of Wolke et al. [65], who describe cyberbullying as a modern tool that supplements traditional forms. Nevertheless, the presence of cyberbullying seems to be an important risk factor for psychological distress and suicidality, especially in adolescents with intensive use of SNS [44]. As a consequence, the combination of PIU, especially the heavy use of SNS, and the presence of cyber-victimization should be specifically assessed.

To our knowledge, no study to date has directly compared the prevalence of PIU in adolescent CAP-IP with a matched control group of nonreferred peers using the same diagnostic instrument. Furthermore, relatively few studies have examined the relationship of PIU and psychiatric comorbidities in the context of inpatient treatment. Our data thus add important new perspectives to the literature. Nevertheless, some limitations should be taken into account. First, the proposed cut-off values for the CIUS [49, 50] have not been empirically determined for adolescents and have been challenged by recent publications [24]. However, using these cut-off values, prevalence figures in our school sample were very close to previous findings gathered in large populational samples from Germany [24, 27], being indicative of the representativeness of our control sample and of the broader generalizability of our findings. Second, there was a time lag of at least a year between the examination of the school sample and data collection among CAP-IP. While a rise of PIU between 2009/2010 and 2011/2012 among European adolescents is described in the literature [66], it is unlikely that this phenomenon explains the large differences between school students and patients in our assessment. Third, only a quantitative comparison between the school sample and the clinical sample was possible because additional data on the mental health of the school students could not be assessed. Fourth, the cross-sectional design of our study provides more of a snapshot than a long-term view of PIU. It is thus important to consider data that suggest that PIU is a volatile syndrome with a tendency to recover over time [67]. Moreover, conclusions on cause and effect of PIU and associated psychopathology cannot be drawn.

## 5. Conclusions

- Problematic and addictive internet use among adolescents undergoing inpatient psychiatric treatment is much more frequent than among their peers in the general population. This suggests that young people with mental health issues have difficulties acquiring balanced, competent media use skills.
- Our study and previous findings point to an association between PIU and certain problem clusters, that is, suicidality, difficulties in establishing stable and consolidated identity, and peer victimization.
- As a consequence, digital media use should be taken into consideration on a routine basis in inpatient treatment and assessment in child and adolescent psychiatry. Assessing PIU and peer victimization may help to identify a high-risk group and thus be an important contribution to the prevention of PIU and to the treatment of affected adolescents in inpatient care.

## Figures and Tables

**Table 1:** Sample characteristics.

Mean age (SD), range	15.1 (1.4)	12–17
Gender		
Male	32	25.2%
Female	83	74.8%
Occupation		
In school	80	72.1%
Apprenticeship	11	9.9%
Other	20	18.0%
Family status		
Parents together	50	45.0%
Parents separated/divorced	55	49.5%
One or both parents deceased	4	3.6%
Data missing	2	1.8%
Psychiatric main diagnosis		
Internalizing disorder (F3, F4, F93)	68	61.3%
Externalizing disorder (F90–F92)	12	10.8%
Eating disorder (F66, F50)	28	25.2%
Other (esp. F1, F98)	3	2.7%
History of suicide attempt	26	23.4%
History of nonsuicidal self-injury	45	40.5%

**Table 2:** Problematic and pathological internet use: comparison of the clinical sample with a sample of nonreferred youth (school sample).

	Clinical sample (*n* = 111)		School sample (*n* = 389)		Cohen's *d*	*t* value	*p* value
	Mean	SD	Mean	SD			
CIUS	14.78	13.75	11.15	8.0	0.32	6.43	<0.001

	%		%		Odds Ratio	95% CI	*p*

Problematic internet use	28.8%		11.0%		3.9	2.3–6.3	<0.001
Pathological internet use	20.7%		3.4%		7.0	3.7–13.3	<0.001

**Table 3:** Sociodemographic data.

	Non- PIU	PIU	*χ* ^2^/*t* value	*p* value
	Mean (SD)	Mean (SD)		
Age	15.2 (1.4)	14.8 (1.5)	1.09	0.28
Family status				
Parents together	34 (43.6%)	16 (51.6%)	1.94	0.38
Parents separated/divorced	40 (51.3%)	15 (48.4%)		
One or both parents deceased	4 (5.1%)	0 (0.0%)		
Gender				
Female	62 (74.7%)	21 (25.3%)	1.99	0.23
Male	17 (60.7%)	11 (39.3%)		

**Table 4:** Clinical data.

	Non-PIU (*n* = 79)	PIU (*n* = 32)	*χ* ^2^/*t* value	*p* value
Psychiatric main diagnosis			4.56	0.10
Externalizing disorder	45 (8.9%)	23 (15.6%)		
Internalizing disorder	7 (57.0%)	5 (71.9%)		
Eating disorder	24 (30.4%)	4 (12.5%)		
History of suicide attempt	11 (13.9%)	15 (46.9%)	13.78	<0.001
Nonsuicidal self-injury (NSSI)	30 (38.0%)	15 (46.9%)	0.75	0.39

**Table 5:** Psychometric data.

	Non-PIU (*n* = 79)		PIU (*n* = 32)		Effect size	*F* value	*p* ^*∗*^
	Mean	(SD)	Mean	(SD)			
AIDA							
Diffusion (total score)	104.1	(40.4)	123.6	(41.1)	0.5	8.25	0.005
Discontinuity	48.0	(17.2)	55.5	(19.8)	0.4	5.48	0.021
Incoherence	56.1	(25.3)	68.1	(25.1)	0.5	8.62	0.004
SCID-II (dimensional scores)							
PD cluster A	1.18	(0.19)	1.26	(0.22)	0.4	3.04	0.084
PD cluster B	1.17	(0.13)	1.25	(0.20)	0.5	4.5	0.036
PD cluster C	1.39	(0.27)	1.42	(0.28)	0.1	0.55	0.58
YSR and CBCL (scales with significant group differences shown)							
YSR							
Attention problems (score)	6.8	(3.9)	8.8	(4.3)	0.5	4.20	0.043
YSR TOT	59.7	(27.2)	66.3	(25.3)	0.2	2.24	0.138
CBCL							
Withdrawn/depressed	6.9	(3.2)	9.4	(3.2)	0.8	13.92	0.001
CBCL TOT	57.9	(27.5)	60.6	(20.3)	0.1	0.97	0.329

*p* ^*∗*^: all *p* values after controlling for gender.

**Table 6:** Peer victimization.

Number of experiences of being bullied or of active bullying	Non-PIU		PIU		Effect size	*t*	*p* ^*∗*^
	Mean	(SD)	Mean	(SD)			
Being bullied offline	7.0	(8.5)	12.8	(9.9)	0.7	10.65	0.002
Being bullied online	0.9	(1.5)	2.1	(2.9)	0.8	9.84	0.002
Active bullying offline	0.05	(0.2)	0.2	(0.4)	0.3	2.42	0.123
Active bullying online	0.2	(0.8)	0.7	(1.3)	0.7	3.29	0.073

*p* ^*∗*^: all *p* values after controlling for gender.

## Abbreviations

PMU: Pathological media use
PIU: Pathological internet use
CAP-IP: Child and adolescent psychiatry inpatients
CAP: Child and adolescent psychiatry
NSSI: Nonsuicidal self-injury.
